# Supplementary material for: Assessing the influence of diverse skills on employability outcomes for IT undergraduates
Source: PLoS One. 2026 Apr 10;21(4):e0346571. doi: 10.1371/journal.pone.0346571 (PMC13068345; doi:10.1371/journal.pone.0346571)
Supplement: Attachment — Submitted filename: Response to Reviewers.docx [file pone.0346571.s002.docx]

**Point by point response to the editor-in-chief, and reviewers**

Dear editor-in-chief, and the reviewers,

We would like to express our profound appreciation to the editor-in-chief, and the reviewers for the valuable comments and suggestions made on our manuscript. Your insights have been instrumental in helping us improve the quality and clarity of the paper. We have carefully considered each comment and have revised the manuscript accordingly. All responses and revisions are detailed point by point in the following document.

Please note that the ***line numbers referred to in this document are aligned with the revised manuscript with track changes***.

# Editors' Comments

| **Editor’s comments** | **Response to the comments** |
| --- | --- |
| 1. Please ensure that your manuscript meets PLOS ONE's style requirements, including those for file naming. The PLOS ONE style templates can be found at  <https://journals.plos.org/plosone/s/file?id=wjVg/PLOSOne_formatting_sample_main_body.pdf> and <https://journals.plos.org/plosone/s/file?id=ba62/PLOSOne_formatting_sample_title_authors_affiliations.pdf> | Thank you very much for bringing this to our attention.  In line with the feedback, we have included a revised first page, adhering to the formal journal guidelines. We have altered the names of the supporting information files to fit the recommended format. |
| 2. Please ensure that you refer to Figure 2 in your text as, if accepted, production will need this reference to link the reader to the figure. | We sincerely appreciate bringing this to our attention.  Figure 2 has been referred to in the Measurement assessment model sub section under Results section. ***(line number 678-679)***  *“The results are detailed in Table 1 and Fig 2 show the correlation and the PLS-SEM algorithm resulted model.”* |
| 3. We are unable to open your Supporting Information file [Title Page.docx]. Please kindly revise as necessary and re-upload. | Duly noted, thank you. We have uploaded the title page in PDF format. |
| 4. Please include captions for your Supporting Information files at the end of your manuscript, and update any in-text citations to match accordingly. Please see our Supporting Information guidelines for more information: http://journals.plos.org/plosone/s/supporting-information. | Thank you for bringing this to our attention.  We have included the Supporting Information files at the end of the manuscript.  ***(line number 668)***  *“The data file is provided in S1 appendix.”* |

# Response to Reviewers

| **Reviewers' comments** | **Response to the comments** |
| --- | --- |
| ***Response to Reviewer 01*** | |
| 1. Reword abstract for smoother expression and remove repetition in phrases like “examines the combined effect… instead of looking at them separately.” | We are grateful for your constructive feedback. In response, Abstract has been reworded and replicated phrases removed.    ***(From line number 36-76)***  “*Rapid technological advancements have reshaped the global job market, emphasizing the importance of specialized competencies such as user interface (UI) and user experience (UX) design, alongside technical and interpersonal skills. This study examines how UI/UX skills (UIUX), soft skills (SS), and technical skills (TS) influence the employability (EP) of IT undergraduates in Sri Lanka., addressing a notable gap in existing literature that often examines these competencies in isolation and predominantly within Western contexts. The current study offers a detailed examination of employability determinants in Sri Lanka’s IT sector by incorporating gender as a moderating factor and investigating the mediating roles of self-efficacy (SE) and proficiency levels (LP). The collection of data involved 345 IT undergraduates participating in structured surveys, which were subsequently analyzed using partial least squares structural equation modelling (PLS-SEM). The demonstrate that gender significantly affects the relationship between soft skills and technical skills with employability, underscoring differences in the v assessment of these competencies among different genders. Moreover, the degree of proficiency influences the connection between technical skills and employability, yet it does not play a significant mediating role in the relationship between soft skills and UI/UX employability. Self-efficacy has proven to be a significant mediator across various skill categories UI/UX, soft, and technical highlighting its essential function in converting competencies into career success. This work seeks to add to existing knowledge by tackling the main significant gap of examining the combined effect of UI/UX, soft, and technical skills on employability. This study contributes to the theoretical understanding of employability by presenting an integrated model that elucidates the complex interactions among skills, mediators, and gender within the Sri Lankan IT sector. The results provide actionable insights for educators, policymakers, and industry leaders, advocating for curriculum alignment with industry needs and the promotion of self-efficacy through mentorship and experiential learning.* |
| 2. Improve flow in introduction and avoid repeating the point that “Western contexts dominate existing research.” | We sincerely appreciate your helpful advice. In line with the suggestion to avoid repetitions, we have improved the Introduction of manuscript.  ***(From line number 77-155)***  *“Rapid technological advancements are transforming industries worldwide, fundamentally reshaping the global labor market and driving a significant increase in demand for specialized roles like user interface (UI) and user experience (UX) designers. This shift grants both significant opportunities and challenges for higher education institutions requiring them to ensure graduates hold the essential skills for success in these emerging fields. [1] The designed model proposes determining if IT undergraduate skills affect UI/UX design jobs.*  *IT degrees are in demand in Sri Lanka, reflecting the global move toward digitalization and the expansion of technology-driven businesses [2]. Employability is "the ongoing fulfilment, acquisition, or creation of work through the optimal utilization of competencies" [2]. In addition, communication, problem-solving, and critical thinking are crucial for IT success, according to studies [2]. Employers value practical knowledge, analytical skills, job dedication, communication and IT skills, management acumen, and favorable attitudes [3]. Listening, interpersonal, verbal and written communication, critical thinking, professionalism, creativity, adaptability, professional confidence, job-specific competencies, leadership, and work experience are also important [3]. However, most existing studies examine UI/UX, technical, and soft skills as individual, and are primarily focused on Western contexts. This leaves a significant gap in understanding how these competencies collectively influence employability in Sri Lanka’s IT sector.. Research is required to evaluate the correlation between the competencies of IT students and the specific requirements of UI/UX design positions.*  *The conducted study examines the intersection of UI/UX capabilities, soft skills, and technical skills in Sri Lanka and graduate preparation for UI/UX careers to address gaps in the literature. This study aims to address these gaps by investigating how UI/UX skills, soft skills, and technical skills among Sri Lankan IT undergraduates impact their employability. It also examines the mediating roles of proficiency and self-efficacy, as well as the moderating effect of gender, in shaping career outcomes. This research effort addresses: what is the impact of UI/UX skills, soft skills and technical skills of Sri Lankan undergraduates for employability? The primary objective is to investigate the impact of UI/UX skills, soft skills and technical skills of Sri Lankan undergraduates for employability. Sri Lanka may capitalize on the global need for UI/UX professionals by training a skilled workforce. This will provide valuable insights for institutions seeking to improve their IT education programs. This effort will educate IT undergraduates about UI/UX capabilities, helping them make educational and career decisions aiming to improve the employability of IT graduates in Sri Lanka's expanding UI/UX design sector by addressing existing gaps. This research delineates the precise objectives it seeks to achieve among Sri Lankan undergraduates from both public and private universities. These objectives are closely linked to research issues and direct the study process.*  *This research is anticipated to yield many substantial contributions that might greatly influence skill sets and inform future practices in undergraduate UI/UX employability. Initially, it will offer an in-depth comprehension of the essential skills and characteristics required for IT graduates to excel in UI/UX design positions within the Sri Lankan environment. Secondly, by examining the mediating roles of proficiency and self-efficacy, this research will provide insights into the fundamental mechanisms that facilitate professional success in this domain. Third, by investigating the potential moderating influence of gender, the study will enhance the comprehension of gender dynamics and any inequities within the UI/UX design industry. This research will provide significant data-driven suggestions for stakeholders, including students, educators, and policymakers, aiming to improve IT graduate employability in Sri Lanka's expanding UI/UX design sector.*  *The following subsections are organized as follows. Section two offers an extensive literature survey, integrating significant academic contributions that underpin the theoretical framework of this study. Section three outlines the technique utilized, encompassing the study design, data gathering, and analysis processes. Section four presents the findings derived from the analysis. Section five presents an analysis of this data, clarifying their significance in relation to the current literature. Section six examines the implications of the findings explicitly for IT education. Sections seven and eight outline the study's shortcomings and provide prospects for further research. The paper concludes with section nine, which provides a summary of the primary findings and their broader implications.”* |
| 3. Clarify timeline expressions like: “Data collection was started in 15/11/24…” → “Data were collected from 15 November 2024 to 10 January 2025.” | Thank you very much for bringing this to our attention.  We included the data collection timeline into the   3.4 Data Collection and Analysis **(Line number 639 - 640)**   *“Data was collected via a structured questionnaire featuring closed-ended questions and a 5-point Likert scale from 15 November 2024 to 10 January 2025.”* |
| 4. Clean up discussion section by removing duplicated explanations (e.g., “gender stereotypes...” repeated twice). | We sincerely appreciate your helpful feedback.  In response to the feedback, we have cleaned up the discussion section.  This has been addressed in Comment 7 of reviewer 2 in detail. |
| 5. Integration of Literature   1. Khan, H. S. U., Li, P., et al. (2023)  Use in your paper:  Section 2.4 – Mediating Impact of Self-Efficacy  Use this study to support your argument that creative self-efficacy plays a mediating role between skill possession and work performance outcomes.  Suggested sentence:  “Prior research confirms that self-efficacy, especially in creative or task-specific domains, acts as a bridge between competencies and innovative work behavior in knowledge-driven fields (Khan et al., 2023), aligning with our model’s inclusion of self-efficacy as a mediator.”  2. Khan, H. S. U., Ma, Z., et al. (2023)  Use in your paper:  Methodology or Section 3 – Research Design / Section 5 – Discussion  This study can be cited to justify your use of a moderated mediation SEM model and the psychological variables involved, such as self-efficacy.  Suggested sentence:  “Our structural model, which includes both mediation and moderation effects, follows the precedent of recent psychological studies using similar SEM frameworks to explore layered behavioral outcomes (Khan et al., 2023).”  3. Chughtai, M. S. & Khan, H. S. U. (2023)  Use in your paper:  Section 2 – Literature Review and Section 4 – Results/Discussion  This paper can reinforce your model’s theoretical basis and support discussion around the indirect effects of skills via psychological mediators, particularly in innovation or tech-based job roles.  Suggested sentence:  “Research by Chughtai and Khan (2023) highlights how knowledge-focused leadership and individual psychological traits such as self-efficacy interact in moderated mediation models to explain innovative performance, which closely parallels our findings on the influence of soft and technical skills on employability outcomes.”  4. Yasmin, F., et al. (2024)  Use in your paper:  Section 2.1 – Employability and Skill Sets / Section 5 – Discussion  Use this to connect employability outcomes to productivity gains and HR-driven skill development.  Suggested sentence:  “Yasmin et al. (2024) emphasize that organizational performance is significantly influenced by HRM practices that develop worker skills and productivity, paralleling our argument that employability is driven by skill readiness shaped through targeted education and training.”  5. Haider, S. A., et al. (2023)  Use in your paper:  Section 2.4 – Self-Efficacy / Section 5 – Discussion  Incorporate this to support the role of knowledge-sharing behavior as a catalyst for self-efficacy and performance outcomes.  Suggested sentence:  “Our results on self-efficacy’s mediating role resonate with Haider et al. (2023), who found that knowledge-sharing and individual confidence drive innovation in project-oriented sectors.”  6. Ali, A., et al. (2021)  Use in your paper:  Section 2.1.3 – Technical Skills and Employability  This fits well to reinforce the link between IT skill acquisition and employability in governance or digital transformation roles.  Suggested sentence:  “Ali et al. (2021) found that strong IT governance frameworks rely on innovation-ready technical personnel, underscoring the importance of technical skillsets for public and private sector employability.”  7. Sohail & Ilyas (2018)  Use in your paper:  Section 2.1 – Employability and Career Outcomes  Connect this study to downstream outcomes of employability—namely, workplace retention and engagement.  Suggested sentence:  “Employability not only influences hiring prospects but also long-term outcomes like organizational commitment (Sohail & Ilyas, 2018), highlighting the importance of foundational skill development for career stability.”  8. Sohail & Rehman (2015)  Use in your paper:  Section 2.4 – Self-Efficacy  Support the idea that self-efficacy can buffer against employment-related stress.  Suggested sentence:  “Psychological resilience, such as self-efficacy, has been shown to moderate workplace stress effects and support healthier professional integration (Sohail & Rehman, 2015).”  Tehseen et al. (2024)  Use in your paper:  Section 6 – Implications or Section 8 – Future Research  Use this when addressing the transferability of your model across regions or cultures.  Suggested sentence:  “Future cross-national studies may benefit from frameworks like Tehseen et al. (2024), who examined how entrepreneurial skillsets vary across countries and influence competitive advantage.”  10. Mata et al. (2021)  Use in your paper:  Section 2.1.2 – Soft Skills / Section 5 – Discussion  This supports inclusion of time management as a key soft skill linked to performance outcomes.  Suggested sentence:  “Mata et al. (2021) demonstrate that effective time management significantly enhances individual performance under workplace stress, reinforcing our inclusion of time as a core soft skill for employability.” | Thank you for the comment. We appreciate your suggestions to improve our manuscript, and we have integrated all the relevant suggested articles into our literature review, research design and future implications sections.  Employability and the Growing Landscape of Skill Sets  ***(From line number 196 to 203)***  *“Perceived employability refers to an individual's confidence in their ability to secure and retain employment in a competitive and evolving labor market [24,25]. This confidence is a complex psychological construct shaped by various factors, rather than merely an optimistic perspective. It encompasses an individual's self-assurance in their competencies, expertise, and skills, along with their understanding of how these attributes align with current and future labor market requirements [26,27].In the current dynamic economic landscape, characterized by continual industry evolution and the emergence of new roles, confidence in one's job-seeking abilities is a significant predictor of successfully obtaining and retaining employment.”*  ***(From line number 204 to 214)***  “*Yorke and Knight emphasize the complexities of employability by underscoring the importance of skills, self-efficacy beliefs, metacognition, comprehension, and learning [36]. Employability metrics encompass perceived employability, job performance, and occupational success [37]. Harvey's research underscores the significance of higher education in fostering attitudes conducive to sustained employability and career progression, while also aiding students in acquiring transferable skills [38]. Employability influences not only initial recruitment but also long-term results, including employee retention and engagement.*  *Ultimately, employers are essential as they invest in graduate training programs, provide honest feedback on skill deficiencies, and actively strive to optimize students' potential. Employability is authentically defined and cultivated within this collaborative ecosystem, where employers, institutions, and students are all actively engaged [39].*”  The Influence of UI/UX Skills on Job Marketability  ***(From line number 255 to 259)***  “*Including UI/UX skills into curricula enhances students' employability in the technology sector by augmenting their ability to address real-world challenges [47]. The incorporation of UI/UX skills into educational programs enhances students' employability in the technology sector by augmenting their problem-solving abilities in real-world scenarios*.”  Soft Skills' Significant Effect on UI/UX Employability  ***(From line number 304 to 313)***  “*According to past studies, students can enhance their communication skills, work well with others, solve problems, and adjust to the demands of the UI/UX design industry [49,59]. Consequently, there exists a significant opportunity to examine the intricate relationship between specific soft skills and their impact on the employability of IT undergraduates pursuing UI/UX positions. This study will provide significant insights for curriculum development and skill enhancement initiatives [60]. Studies conducted by Mata et al. (2021) [61] indicate that effective time management significantly reduces workplace stress and enhances individual work performance. Their findings indicate a strong positive correlation between job performance and time management skills, underscoring the significance of time management as a crucial soft skill in the workplace, especially in high-pressure scenarios.”*  Technical Skills' Influence on Job Marketability  ***(From line number 345 to 351)***  “*Technical skills are increasingly essential for employment in positions related to digital transformation and governance. Ali et al. (2021) [65] assert that robust IT governance frameworks necessitate technical personnel equipped for innovation, highlighting the significance of technical skill sets in both public and private sector employment. Their findings underscore the imperative for continuous IT skill enhancement to meet evolving organizational and digital innovation requirements*.”  Moderating Effect of Gender  ***(From line number 382 to 387)***  “*Research indicates that gender and institutional type serve as moderators influencing the relationship between skills and employability [73–76]. The type of institution affects resource accessibility and teaching methodologies that influence proficiency development, while documented gender disparities in self-efficacy and skill application within technology education affect career outcomes. The integration of these moderators into the model facilitates a more nuanced comprehension of employability pathways.”*  Mediating Impact of Self-Efficacy  ***(From line number 468 to 471)***  “*Enstroem and Schmaltz (2024) [95] introduce a 'work-readiness hierarchy' that differentiates between transversal skills and employability outcomes, highlighting the significance of psychological mediators, such as self-efficacy, in linking skill acquisition to employment success.*”  ***(From line number 481 to 484)***  “*Enstroem and Benson (2024) [98] demonstrate that enterprise education interventions foster authentic self-efficacy centered on confidence in unfamiliar tasks, rather than merely correlating with skill acquisition, highlighting the necessity of assessing self-efficacy as a separate construct indicative of task-specific confidence.”*  ***(From line number 489 to 498)***  “*The incorporation of self-efficacy as a mediator in presented model aligns with prior studies indicating that self-efficacy, especially in creative or task-specific areas, acts as a conduct between competencies and innovative work behavior in knowledge-intensive sectors [99]. Consequently, it is deemed rational to presume that students' attitudes and motivations concerning employability will be affected by their self-evaluation of skills and competencies [100,101]. Students can reinforce the connection between theoretical concepts and tangible, real-world applications by acquiring practical experience through workplace engagements during their undergraduate education. This enhances their confidence in undertaking internship assignments and elevates their overall preparedness for future professional pursuits [102].”*  ***(From line number 531 to 534)***  *“Benson and Enstroem's (2017) [107] findings that professional skill development and self-confidence co-evolve within undergraduate curricula, facilitating the integration of related constructs such as technical skills and self-efficacy, while acknowledging their dynamic interaction.”*  Research Design  ***(From line number 617 to 624)***  “*Furthermore, the model's theoretical framework is grounded in work readiness and social cognitive theories, emphasizing the essential function of psychological mediators in transforming skill acquisition into employability results, especially in innovation. Nevertheless, these skills are effectively utilized to enhance employability through psychological principles such as self-efficacy. Self-efficacy quantifies an individual's confidence in their ability to apply their skills in novel and challenging circumstances, which is vital in swiftly evolving technological landscapes where adaptability and innovation are imperative [119].”*  The following is a list of DOIs for the citations that were added in the lines above.  24. Räty H, Hytti U, Kasanen K, Komulainen K, Siivonen P, Kozlinska I. Perceived employability and ability self among Finnish university students. European Journal of Psychology of Education [Internet]. 2020;35(4):975–93. Available from: <https://doi.org/10.1007/s10212-019-00451-7>  25. Niu Y, Hunter-Johnson Y, Xu X, Liu T. Self-Perceived Employability and Subjective Career Success: Graduates of a Workforce Education and Development Program. The Journal of Continuing Higher Education [Internet]. 2019;67(2–3):55–71. Available from: <https://doi.org/10.1080/07377363.2019.1660843>  26. Monteiro S, Ferreira JA, Almeida LS. Self-perceived competency and self-perceived employability in higher education: the mediating role of career adaptability. J Furth High Educ [Internet]. 2020;44(3):408–22. Available from: <https://doi.org/10.1080/0309877X.2018.1542669>  27. Turner NK. Development of self-belief for employability in higher education: ability, efficacy and control in context. Teaching in Higher Education [Internet]. 2014;19(6):592–602. Available from: <https://doi.org/10.1080/13562517.2014.901951>  36. Knight PT, Yorke M. Employability and Good Learning in Higher Education. Teaching in Higher Education [Internet]. 2003;8(1):3–16. Available from: <https://doi.org/10.1080/1356251032000052294>  37. Behle H. Students’ and graduates’ employability. A framework to classify and measure employability gain. Policy Reviews in Higher Education [Internet]. 2020;4(1):105–30. Available from: <https://doi.org/10.1080/23322969.2020.1712662>  39. Arranz N, Arroyabe MF, Sena V, Arranz CFA, de Arroyabe JCF. University-enterprise cooperation for the employability of higher education graduates: a social capital approach. Studies in Higher Education [Internet]. 2022;47(5):990–9. Available from: <https://doi.org/10.1080/03075079.2022.2055323>  47. Guerino GC, Balancieri R, Leal GCL, Prikladnicki R. Towards the UX Support to Software Startups: On the relationship of professional expertise and UX work in the Brazilian scenario. Journal of Systems and Software [Internet]. 2025 Jan;219:112246. <https://doi.org/10.1016/j.jss.2024.112246>  59. Finch DJ, Hamilton LK, Baldwin R, Zehner M. An exploratory study of factors affecting undergraduate employability. Education and Training. 2013;55(7):681–704. <https://doi.org/10.1108/ET-07-2012-0077>  60. Teng W, Ma C, Pahlevansharif S, Turner JJ. Graduate readiness for the employment market of the 4th industrial revolution: The development of soft employability skills. Education and Training. 2019 Jul 12;61(5):590–604. <https://doi.org/10.1108/ET-07-2018-0154>  61. Nuno Mata M, Arslan Haider S, Xavier Rita J. Special Issue 6, 2021 1 Strategic Management & Decision Process. Academy of Strategic Management Journal [Internet]. 2021 [cited 2025 Jul 1];20. Available from: <https://www.abacademies.org/articles/the-relationship-between-time-management-work-stress-and-work-performancea-quantitative-study-in-portugal-12182.html>  65. Ali A, Al-Sulaiman FA, Al-Duais INA, Irshad K, Malik MZ, Shafiullah M, et al. Renewable Portfolio Standard Development Assessment in the Kingdom of Saudi Arabia from the Perspective of Policy Networks Theory. Processes [Internet]. 2021;9(7). <https://doi.org/10.3390/pr9071123>  73. Gokuladas VK. Predictors of Employability of Engineering Graduates in Campus Recruitment Drives of Indian Software Services Companies. International Journal of Selection and Assessment [Internet]. 2011;19(3):313 9. <https://doi.org/10.1111/j.1468-2389.2011.00560.x>  74. Scandurra R, Kelly D, Fusaro S, Cefalo R, Hermannsson K. Do employability programmes in higher education improve skills and labour market outcomes? A systematic review of academic literature. Studies in Higher Education. 2024;49(8):1381–96. <https://doi.org/10.1080/03075079.2023.2265425>  75. Chiu SY, Chuang HL. Employability and Wage Compensation in an Asian Economy: Evidence for Female College Graduates in Taiwan. Emerging Markets Finance and Trade [Internet]. 2016 Apr 2;52(4):853–68. Available from: <https://doi.org/10.1080/1540496X.2015.1117844>  76. Adhikari A, Vethman S, Vos D, Lenz M, Cocu I, Tolios I, et al. Gender mobility in the labor market with skills-based matching models. AI and Ethics. 2023 Jul 17;4:163–7. <https://doi.org/10.48550/arXiv.2307.08368>  95. Enstroem R, Schmaltz R. Striking gold: navigating the education massification maze for work readiness. Journal of Work-Applied Management. 2024 Sep 13;16(2):184–99. <https://doi.org/10.1108/JWAM-10-2023-0100>  98. Enstroem R, Benson L. Enterprise education in undergraduate business programmes advances students’ negotiating competence and self-confidence. Education + Training [Internet]. 2024 Jan 1;66(1):54–69. Available from: <https://doi.org/10.1108/ET-01-2022-0009>  99. Khan HS ud din, Li P, Chughtai MS, Mushtaq MT, Zeng X. The role of knowledge sharing and creative self-efficacy on the self-leadership and innovative work behavior relationship. Journal of Innovation and Knowledge. 2023 Oct 1;8(4)  100. Wujema BK, Mohd Rasdi R, Abu Samah B, Abdullah ALK, Abdul Aziz MF. Examining the Mediating Effect of Self-efficacy on the Relationship between Work Experience, ICT Acceptance and Employability among Undergraduate Students in Nigerian Universities. International Journal of Academic Research in Business and Social Sciences. 2020 Mar 27;11(3). <https://doi.org/10.3390/su14084660>  101. White M, Becker J, du Plessis M. Unintended Positive Consequences of Development Centres in University Graduates. Front Psychol. 2021 Dec 1;12. <https://doi.org/10.3389/fpsyg.2021.775377>  102. Oberman W, Hunt I, Taylor RK, Morrisette S. Internships and occupational self-efficacy: Impact and gender differences. Journal of Education for Business [Internet]. 2021 Oct 3;96(7):424–34. Available from: <https://doi.org/10.1080/08832323.2020.1848768>  107. Benson L, Enström R. Deliberate institutional differentiation through graduate attributes: Implementing professional skills at MacEwan School of Business. Journal of Management Development. 2017;36(6):817–27. <https://doi.org/10.1108/JMD-08-2016-0148>  119. Chughtai MS, Khan HS ud din. Knowledge oriented leadership and employees’ innovative performance: a moderated mediation model. Current Psychology [Internet]. 2024;43(4):3426–39. Available from: <https://doi.org/10.1007/s12144-023-04502-7> |
| ***Response to Reviewer 02*** | |
| 1. Your structural model includes three antecedent skill constructs (UI/UX, soft, technical), two mediators (self-efficacy and proficiency), and gender interactions. Several latent variables (e.g., Soft Skills vs Soft-Skill Proficiency, Technical Skills vs Technical-Skill Proficiency, and Skills vs Self-Efficacy) overlap conceptually and empirically, leading to very high intercorrelations (HTMT > 0.90) and collinearity (VIF > 7).   For instance, measuring ‘I can communicate effectively’ (soft skill) alongside ‘I am highly proficient in communication’ (proficiency) will produce almost identical responses, which means that the mediator-test (soft skills → proficiency → employability) becomes meaningless.   - Simplify by merging redundant constructs.   For instance, combine Soft Skills and their corresponding proficiency items into a single Soft-Skill Competence factor. Likewise, merge Technical Skills with Technical-Skill Proficiency, and ensure Self-Efficacy items capture confidence in performing tasks beyond current ability. Reducing the number of latent variables will alleviate identification issues and produce more stable path estimates. | We value the recognition of its significance in the work readiness literature. We acknowledge the apprehensions regarding possible conceptual redundancy and significant intercorrelations among certain latent variables, especially the interaction between skill constructs, self-efficacy, and proficiency. The recommendation to reassess these relationships is acknowledged.  Our framework is intended to highlight the distinct contributions and interactions of various constructs. Each represents a different aspect of an individual's readiness.  Our conceptualization of skills, proficiency, and self-efficacy as separate but interconnected constructs is based on a significant amount of educational literature.  These constructs encompass various aspects of an individual's abilities and perceptions.   - **Skills** denote the intrinsic, fundamental abilities an individual possesses, encapsulating the knowledge, competencies, and talents they have acquired. - **Proficiency** signifies the exhibited degree of expertise or mastery in the practical application of skills. - **Self-Efficacy** refers to an individual's conviction in their ability to perform actions required to achieve particular performance outcomes.   Bandura's (1978) work on self-efficacy established it as a psychological construct separate from skills and proficiency. Bandura argued that people may possess the necessary skills but lack the confidence to apply them, demonstrating the importance of measuring these constructs separately. Mandal et al. (2025) and Iso-Ahola (2024) demonstrate that, while skills serve as the foundation, proficiency reflects the level of skill application, and self-efficacy mediates whether individuals use their skills effectively. Further research in language learning and employability emphasizes the importance of measuring each construct separately to accurately assess development and outcomes.  As you suggested, we examined alternative model specifications in which "skills" and "proficiency" were regarded as a singular construct. This alternative model produced results that were analytically valid, demonstrating comparable or even reduced challenges regarding model fitness and interpretation. [Resulted values are attached for further reference]  However, attributing these solely to conceptual overlaps among distinct constructs may be an oversimplification.  Instead of merging distinct constructs, our method recognizes the strong correlations while meticulously analyzing the individual contributions of each variable within the model.  Additionally, we have analyzed the importance of the path coefficients to comprehend the relative strength of the relationships, despite the existence of high intercorrelations.  Our main emphasis has been on illustrating the distinct predictive validity of each construct, especially in mediating the connection between skills and employability. The observation that the proposed merged models produced even less favorable outcomes reinforces our existing conceptualization.  We contend that combining these constructs into a single measure, even if analytically correlated, would obscure the subtle pathways we seek to investigate, ultimately reducing the usability and theoretical richness of our findings.  The following literature discusses the conceptualization of skills, proficiency, and self-efficacy as separate but interrelated constructs.   - Bandura, A. (1978). Self-efficacy: Toward a unifying theory of behavioral change. *Advances in Behaviour Research and Therapy*, *1*(4), 139–161. <https://doi.org/10.1016/0146-6402(78)90002-4> - Mandal, S., Mukhopadhyay, P., & Jana, S. (2025). Integrating CAVAL Competencies and the Five-Factor Model for Library and Information Science Professionals in Higher Education. *New Review of Academic Librarianship*, 1–21. <https://doi.org/10.1080/13614533.2025.2491320> - Iso-Ahola, S. E. (2024). A theory of the skill-performance relationship. *Frontiers in Psychology*, *15*. <https://doi.org/10.3389/fpsyg.2024.1296014> - Wang, J., Wang, J., & Wei, R. (2025). Is language proficiency linked to intercultural communicative competence? *Language, Culture and Curriculum*, 1–22. <https://doi.org/10.1080/07908318.2025.2507604> - Soproni Z. Employability Skills: Rethink Your Learning. GJSD [Internet]. 2023 Oct. 25 [cited 2025 Jul. 13];3(2):53-65. Available from: <https://gjsd.gile-edu.org/index.php/home/article/view/6-soproni-53-65> - Arribas-Aguila, D., Castaño, G., & Martínez-Arias, R. (2024). A Systematic Review of Evidence-based General Competency Models: Development of a General Competencies Taxonomy. *Journal of Work and Organizational Psychology, 40* (2), 61 - 76. <https://doi.org/10.5093/jwop2024a6> - Suyitno, S., Saputro, I. N., Jatmoko, D., Widiyono, Y., Purwoko, R. Y., Abdillah, F., Setuju, S., & Hermawan, Y. (2025). The Effect of Work-Based Learning on Employability Skills: The role of Self-Efficacy and Vocational Identity. *European Journal of Educational Research*, *14*(1), 309–321. <https://doi.org/10.12973/eu-jer.14.1.309> - Sulong, N., & Mahfar, M. (2024b). A Conceptual Paper of Career Self-Efficacy and Career Readiness among Secondary School Students. *International Journal of Academic Research in Business and Social Sciences*, *14*(11). <https://doi.org/10.6007/ijarbss/v14-i11/23457> - Borg, J., Scott-Young, C. M., & Borg, N. (2023). Early career project managers’ work readiness: Adopting a self-efficacy lens. *International Journal of Project Management*, *41*(2), 102454. <https://doi.org/10.1016/j.ijproman.2023.102454> - Borg, J., Scott‐Young, C. M., & Bartram, T. (2025). A Review of Graduate Work Readiness Literature: A conceptual exploration of the implications for HRM research and practice. *Asia Pacific Journal of Human Resources*, *63*(2). [https://doi.org/10 .1111/1744-7941.70012](https://doi.org/10%20.1111/1744-7941.70012) - Lim, W.M. (2024), "A typology of validity: content, face, convergent, discriminant, nomological and predictive validity", *Journal of Trade Science*, Vol. 12 No. 3, pp. 155-179. <https://doi.org/10.1108/JTS-03-2024-0016> - Ringle, Christian M., Wende, Sven, & Becker, Jan-Michael. (2024). SmartPLS 4. Bönningstedt: SmartPLS. Retrieved from <https://www.smartpls.com> |
| 2. Several constructs are treated as unidimensional despite comprising heterogeneous subdomains (e.g., eight soft-skill dimensions combined into one factor; five UI/UX subdomains collapsed without hierarchical testing). Some subscales have only two indicators, which raises questions about identification and reliability.   Conduct a standalone factor analysis to establish that each latent variable has at least three coherent indicators or is a justified two-indicator factor.  For broad constructs (e.g., Soft Skills), either retain only the most relevant subdomains (e.g., communication, teamwork, problem-solving) or adopt a second-order factor structure: first demonstrate that each subdimension is unidimensional, then confirm that they load onto a higher-order Soft-Skill Competence factor.  All variables are self-reported in one sitting. As a result, method variance likely inflates intercorrelations.  Moreover, causal language (Skill A influences Employability through Self-Efficacy’) is used despite cross-sectional data. The very large path coefficient from Technical Skills to Employability ( ≈ 0.89) may partly reflect common-method bias rather than a true causal effect. | We acknowledge the reviewer’s valid concerns regarding the treatment of broad constructs as unidimensional and the presence of two-indicator factors.  We treat some broad constructs as unidimensional because the dimensions cover the full spectrum of the underlying factor, representing a single coherent latent trait.  Under essential unidimensionality, a strong general factor explains most variance across heterogeneous subdomains. Recent psychometric research supports this approach.   - Reise, S. P., Bonifay, W. E., & Haviland, M. G. (2012). Scoring and Modeling Psychological Measures in the Presence of Multidimensionality. *Journal of Personality Assessment*, *95*(2), 129–140. <https://doi.org/10.1080/00223891.2012.725437> - Liu, X., Cao, P., Lai, X., Wen, J., & Yang, Y. (2022). Assessing Essential Unidimensionality of Scales and Structural Coefficient Bias. *Educational and Psychological Measurement*, *83*(1), 28-47. <https://doi.org/10.1177/00131644221075580>   Our initial EFA revealed that, while multiple dimensions existed at the specific level, a strong first-order factor appeared for each broad category, indicating a dominant underlying factor that explained a significant proportion of the variance across the subdomains. Sub-dimensions existed, but their high intercorrelations suggested treating them as a single, higher-order construct was conceptually and empirically justified in our model. When we attempted to implement a complete second-order factor structure, the models frequently struggled with identification. Our initial reliability analyses for these two-indicator constructs consistently revealed acceptable levels of internal consistency, lending empirical support to their coherence.  We conducted preliminary analyses, Confirmatory Factor Analysis,  Our findings indicated that a rigid observance to these suggestions did not consistently yield analytically superior or theoretically more meaningful models for our specific dataset and research context.  Our reliability analyses for these specific two-indicator constructs consistently demonstrated acceptable internal consistency, supporting their coherence. Attempting to force additional indicators or removing one to create a single-item measure would have either weak conceptual accuracy or significantly compromised reliability.  Therefore, while we appreciate the suggestion, our prior analyses led us to the current model specification as analytically supported and theoretically meaningful representation for our data. |
| 3. In the revised manuscript, explicitly acknowledge CMV as a limitation; perform a post hoc diagnostic (e.g., Harman’s one-factor test or inclusion of a common latent factor in PLS-SEM) and report the proportion of variance attributable to method effects. | Thank you for pointing out this area for improvement. In the revised manuscript's Limitations of the Study section, we explicitly acknowledge CMV as a limitation, explaining how our cross-sectional, self-report data may contribute to inflated correlations.  ***(From line number 934 to 940)***  *“This study is constrained by its reliance on cross-sectional, self-reported data collected during a single session which may result in common method variance (CMV). This bias could amplify correlations and path coefficients possibly inflating the connection between technical Skills and employability. The analyses provide valuable insights into the absence of control for CMV and the challenge in establishing causality limit the interpretation of the findings. Future investigations should employ longitudinal designs or multi-source data to mitigate common method variance.”*  Common Latent Factor PLS-SEM:  The incorporation of a latent variable that is applied to all main measures in the model. The substantive path coefficients generally underwent minor changes when we compared the standardized path coefficients with and without the CLF. Additionally, the significance levels of the core relationships remained largely unchanged. This implies that the pattern or significance of our theoretical relationships are not fundamentally altered by the existence of some method variance.   - Podsakoff, P. M., MacKenzie, S. B., Lee, J.-Y., & Podsakoff, N. P. (2003). Common method biases in behavioral research: A critical review of the literature and recommended remedies. *Journal of Applied Psychology, 88*(5), 879–903. <https://doi.org/10.1037/0021-9010.88.5.879> - Fuller, C. M., Simmering, M. J., Atinc, G., Atinc, Y., & Babin, B. J. (2015). Common methods variance detection in business research. *Journal of Business Research*, *69*(8), 3192–3198. <https://doi.org/10.1016/j.jbusres.2015.12.008> |
| 4. Rephrase all causal claims as associations (e.g., technical skills are associated with perceived employability’). | Thank you for the comment.  In the revised manuscript, we have reviewed and rephrased all instances of causal language. This rephrasing will be applied consistently throughout the Abstract, Introduction, Results, and Discussion sections. |
| 5. The literature review summarizes existing studies without critically comparing theoretical frameworks or engaging deeply with regional research.  Hypotheses are stated based on generic claims rather than being grounded in specific empirical findings.  - Reorganize the review into clear themes: - Definitions and measures of employability (e.g., distinguish between perceived employability and actual placement outcomes). - Empirical evidence on UI/UX, soft, and technical skills, drawing on global and South Asian studies. - Mediators and moderators, explicitly linking each hypothesis to at least two prior studies. | We sincerely appreciate your advice.  In line with the insight, we have improved the literature review.   - Reorganize the review into clear themes:   Literature review reorganized to the following order;  Literature Review   - Employability and the Growing Landscape of Skill Sets - The Direct Impact of Different Skill Domains on Employability   - The Influence of UI/UX Skills on Job Marketability   - Soft Skills' Significant Effect on UI/UX Employability   - Technical Skills' Influence on Job Marketability - Moderating the Relationship Between Skill and Employability   - Moderating Effect of Gender - Mediating the Relationship Between Skill and Employability   - Mediating Impact of Level of Proficiency   - Mediating Impact of Self-Efficacy - Definitions and measures of employability   ***(From line number 553 to 570)***  *“Conceptualizing and measuring perceived employability varies widely across studies. This study assesses perceived employability by assessing self-confidence, perception of external job opportunities, field reputation, and institutional prestige. The dimensions are operationalized using validated scales [108]. Self-belief measures personal employability, while external labor market awareness measures job availability and quality. This multidimensional approach uses Likert-scale items to assess perceived employability by measuring cognitive and affective perceptions. According to Tomlinson (2008) [109], students increasingly view academic qualifications as necessary but insufficient, emphasizing the need to add skills and experience to compete. This study measures perceived employability using self-confidence in skills, labor market awareness, and academic and institutional reputation. Recent research emphasizes the importance of digital skills in employability perceptions. Tee et al. (2024) [110] list “information and data literacy,” “problem-solving,” and “digital content creation” as top demands, with “communication and collaboration” emerging as a major skill gap that affects employability. These findings support assessing technical and interpersonal abilities in employability frameworks. These perspectives inform a solid, comprehensive measurement framework that captures cognitive, skill-based, and contextual aspects of perceived employability, making it relevant to current labor market realities and the IT sector's dynamic skill demands.”*  The following is a list of DOIs for the citations that were added in the lines above.  108. Rothwell A, Arnold J. Self-perceived employability: Development and validation of a scale. Personnel Review. 2007;36(1):23–41. <https://doi.org/10.1108/00483480710716704>  109. Tomlinson M. Graduate employability: A review of conceptual and empirical themes. Vol. 25, Higher Education Policy. 2012. p. 407–31. <https://doi.org/10.1057/hep.2011.26>  110. Tee PK, Song BL, Ho MK, Wong LC, Lim KY. Bridging the gaps in digital skills: Employer insights on digital skill demands, micro-credentials, and graduate employability. Journal of Infrastructure, Policy and Development. 2024;8(9). <https://doi.org/10.24294/jipd.v8i9.7313>   - Empirical evidence on UI/UX, soft, and technical skills, drawing on global and South Asian studies.   We have improved the literature as suggested with practical evidence on UI/UX, soft, and technical skills, drawing on global and South Asian studies.  UIUX skills  **(line number 255-259)**  *“Including UI/UX skills into curricula enhances students' employability in the technology sector by augmenting their ability to address real-world challenges [47]. The incorporation of UI/UX skills into educational programs enhances students' employability in the technology sector by augmenting their problem-solving abilities in real-world scenarios.”*  Soft skills  ***(line number 304-313)***  *“According to past studies, students can enhance their communication skills, work well with others, solve problems, and adjust to the demands of the UI/UX design industry [49,59]. Consequently, there exists a significant opportunity to examine the intricate relationship between specific soft skills and their impact on the employability of IT undergraduates pursuing UI/UX positions. This study will provide significant insights for curriculum development and skill enhancement initiatives [60]. Studies conducted by Mata et al. (2021) [61] indicate that effective time management significantly reduces workplace stress and enhances individual work performance. Their findings indicate a strong positive correlation between job performance and time management skills, underscoring the significance of time management as a crucial soft skill in the workplace, especially in high-pressure scenarios.”*  Technical skills  ***(line number 345-351 )***  *“Technical skills are increasingly essential for employment in positions related to digital transformation and governance. Ali et al. (2021) [65] assert that robust IT governance frameworks necessitate technical personnel equipped for innovation, highlighting the significance of technical skill sets in both public and private sector employment. Their findings underscore the imperative for continuous IT skill enhancement to meet evolving organizational and digital innovation requirements.”*   - Mediators and moderators, explicitly linking each hypothesis to at least two prior studies.   We have improved the literature explicitly including prior studies for each mediator and moderator hypothesis.  Moderator: Gender  ***(line number 382 - 387)***  *“Research indicates that gender and institutional type serve as moderators influencing the relationship between skills and employability [73–76]. The type of institution affects resource accessibility and teaching methodologies that influence proficiency development, while documented gender disparities in self-efficacy and skill application within technology education affect career outcomes. The integration of these moderators into the model facilitates a more nuanced comprehension of employability pathways.”*  Mediator: Self-efficacy  ***(line number 482 - 487)***  *“Enstroem and Schmaltz (2024) [95] introduce a 'work-readiness hierarchy' that differentiates between transversal skills and employability outcomes, highlighting the significance of psychological mediators, such as self-efficacy, in linking skill acquisition to employment success.”*  ***(line number 481 - 484)***  *“Enstroem and Benson (2024) [98] demonstrate that enterprise education interventions foster authentic self-efficacy centered on confidence in unfamiliar tasks, rather than merely correlating with skill acquisition, highlighting the necessity of assessing self-efficacy as a separate construct indicative of task-specific confidence.”*  ***(line number 489 - 494)***  *“The incorporation of self-efficacy as a mediator in presented model aligns with prior studies indicating that self-efficacy, especially in creative or task-specific areas, acts as a conduct between competencies and innovative work behavior in knowledge-intensive sectors [99]. Consequently, it is deemed rational to presume that students' attitudes and motivations concerning employability will be affected by their self-evaluation of skills and competencies [100,101].”*  ***(line number 531 - 534)***  *“Benson and Enstroem's (2017) [107] findings that professional skill development and self-confidence co-evolve within undergraduate curricula, facilitating the integration of related constructs such as technical skills and self-efficacy, while acknowledging their dynamic interaction.”* |
| 6. The manuscript’s current laundry list of relationships (three skill types, two mediators, two moderators) creates a disjointed narrative, and the practical implications are very general.   After merging overlapping constructs and ensuring discriminant validity, focus on the two or three strongest antecedents that remain distinct.  For example, if multicollinearity prevents distinguishing Technical Skills from Self-Efficacy, consider reporting a combined Technical-Self-Efficacy factor. Present a simplified PLS-SEM with clearer, theory-driven paths (e.g., UI/UX skills → Self-Efficacy → Employability, Soft-Skill Competence → Employability) and, if you wish to explore moderation, run multi-group analyses (e.g., by gender). | We thank you for your insightful comment regarding the complexity of our model and the clarity of our narrative and practical implications.  Our current framework encompasses three skill types, two distinct mediators, and one moderator which is purposely structured based on specific foundational theories or prominent frameworks. Each construct and its proposed relationship are grounded in established literature, as detailed in our theoretical development section.  Regarding the mediators, we acknowledge the reviewer's concern about potential overlap.  While the number of relationships might appear extensive, each component plays a theoretically distinct and crucial role in comprehensively understanding the multifaceted nature of work readiness. This has been addressed in Comment 1 of reviewer 2 in detail.  We have revised the discussion section. We have now highlighted the unique theoretical contributions of each mediator and skill.  ***(line numbers 892-911)***  *“Previous research has emphasized the co-evolution of competence and confidence in educational and occupational development [98,107]. The significance of educational interventions that concurrently enhance students' technical skills and cultivate their self-efficacy in tackling new and challenging tasks is underscored by the distinct yet complementary functions of these elements. The study by Chughtai and Khan (2024) [119] indicates that knowledge-oriented leadership influences employees' innovative performance through mediators such as work engagement and knowledge-sharing behavior, with creative self-efficacy acting as a significant moderator. Their moderated mediation model posits that psychological variables both mediate and amplify the impact of knowledge and skills on creative outcomes within the IT sector. This closely mirrors the findings, demonstrating that employability and self-efficacy are mediated by self-efficacy, suggesting that confidence enhances the translation of skills into tangible career advantages.*  *The findings of Haider et al. (2023) [133], which indicate that knowledge sharing, and personal confidence are crucial mechanisms fostering innovation in project-based sectors, align closely with the results demonstrating that self-efficacy mediates the relationship between employability and soft skills among IT undergraduates. Haider and colleagues found that the influence of ambidextrous leadership on creative work behavior is mediated by knowledge sharing. This underscores the significance of self-confidence and belief in one's capabilities in employing communication and interpersonal skills to excel in dynamic, technologically advanced environments.”* |
| 7. Finally, revise the Discussion to offer targeted recommendations, such as integrating enterprise-education modules (Enstroem & Benson, 2024) to build self-efficacy through hands-on projects, or leveraging adaptive learning platforms (Enstroem & Schmaltz, 2024) to scale soft-skill development in large classes. | Thank you for your comment. We appreciate your suggestions to improve the research paper, and we have integrated the suggested relevant papers into our discussion.  ***(From line number 773 to 782)***  *“The findings of Mata et al. (2021) [61] indicate that time management correlates with improved performance outcomes, thereby justifying our classification of time management as an essential soft skill. Efficient time management is essential for employability as it mitigates stress and enhances productivity. Efficient time management allows individuals to organize and oversee their tasks [65]. It assists individuals in organizing and executing tasks efficiently, thereby enhancing productivity and reducing stress levels [121]. Communication, adaptability, collaboration, and analytical reasoning are essential determinants of employability [59]. These results underscore the significance of soft skills in securing employment. Career success is significantly influenced by employability skills, particularly self-management and job performance [122].”*  ***(From line number 805 to 814)***  “*Misra and Khurana (2017) [126] underscored the necessity for advanced technical competencies within employability frameworks, defining technical skills as one of six critical skill sets requisite for IT professionals to fulfill industry requirements. Puwakgahawela et al. (2023) [127] identify significant discrepancies between employers' expectations and graduates' technical competencies, underscoring the imperative for colleges to modify their curricula to align with industry requirements. Moreover, study demonstrate that technical skills are crucial for career progression, employment opportunities, and adaptability in rapidly changing IT landscapes. All peer-reviewed studies concur that technical skills are vital for the employability of IT undergraduates and that continuous curriculum updates and practical training are required to align with industry standards.*  ***(From line number 834 to 850)***  *“Carter (2011) [128] and Groeneveld, Becker, and Vennekens (2020) [113] assert that soft skills such as communication, teamwork, adaptability, and problem-solving are increasingly vital for the employability of IT graduates. Studies demonstrate that female students often exhibit heightened confidence in soft skills, potentially enhancing their employability by counteracting gender biases in technical domains [71,80,129]. These skills assist recent female graduates in maneuvering through workplace dynamics and overcoming structural barriers. Conversely, soft skills may hold lesser significance for the employability of male students compared to technical skills. This gender moderation indicates that to enhance comprehensive career readiness, employability programs must emphasize the cultivation of soft skills alongside technical training, especially for female IT students. Gender, however, influences this relationship: while core technical skills do not significantly differ between male and female students, the pervasive gender biases in the tech industry frequently hinder female graduates from converting these skills into employment opportunities [71,77,78]. Targeted interventions are essential to enhance the technical self-efficacy and workplace acceptance of female IT students, as these biases can undermine women's confidence and acknowledgment in technical positions [130] . Consequently, while technical* *skills are crucial, gendered social and organizational factors affect their employability.”*  ***(From line number 892 to 911)***  *“Previous research has emphasized the co-evolution of competence and confidence in educational and occupational development [98,107]. The significance of educational interventions that concurrently enhance students' technical skills and cultivate their self-efficacy in tackling new and challenging tasks is underscored by the distinct yet complementary functions of these elements. The study by Chughtai and Khan (2024) [119] indicates that knowledge-oriented leadership influences employees' innovative performance through mediators such as work engagement and knowledge-sharing behavior, with creative self-efficacy acting as a significant moderator. Their moderated mediation model posits that psychological variables both mediate and amplify the impact of knowledge and skills on creative outcomes within the IT sector. This closely mirrors the findings, demonstrating that employability and self-efficacy are mediated by self-efficacy, suggesting that confidence enhances the translation of skills into tangible career advantages.*  *The findings of Haider et al. (2023) [133], which indicate that knowledge sharing, and personal confidence are crucial mechanisms fostering innovation in project-based sectors, align closely with the results demonstrating that self-efficacy mediates the relationship between employability and soft skills among IT undergraduates. Haider and colleagues found that the influence of ambidextrous leadership on creative work behavior is mediated by knowledge sharing. This underscores the significance of self-confidence and belief in one's capabilities in employing communication and interpersonal skills to excel in dynamic, technologically advanced environments.”*  The DOIs for the citations added in the above-mentioned lines are listed below,  71. Honnamane PS, Sreekanth K, Girish GP. The moderating role of gender in the relationships among job characteristics, perceived organizational support, engaging leadership, and work engagement in the Indian IT sector. International Journal of Advanced and Applied Sciences. 2024 Aug 1;11(8):51–6. <https://doi.org/10.21833/ijaas.2024.08.006>  77. Lu L. Effects of demographic variables perceived spousal support, and gender role attitudes on taiwanese women’s employability. J Career Dev. 2011 Jun;38(3):191–207. <https://doi.org/10.1177/0894845309360415>  78. Santos-Jaén JM, Iglesias-Sánchez PP, Jambrino-Maldonado C. The role of gender and connections between entrepreneurship and employability in higher education. International Journal of Management Education. 2022 Nov 1;20(3). <https://doi.org/10.1016/j.ijme.2022.100708>  80. Gao Yanxia. Gender Differences in Career Counseling:Influencing Factors and Coping Strategies. Academic Journal of Humanities & Social Sciences. 2023;6(18). <https://doi.org/10.25236/AJHSS.2023.061822>  112. Bedoya-Guerrero A, Basantes-Andrade A, Rosales FO, Naranjo-Toro M, León-Carlosama R. Soft Skills and Employability of Online Graduate Students. Educ Sci (Basel). 2024 Aug 1;14(8). <https://doi.org/10.3390/educsci14080864>  113. Groeneveld W, Becker BA, Vennekens J. Soft Skills: What do Computing Program Syllabi Reveal About Non-Technical Expectations of Undergraduate Students? [Internet]. 2020 May [cited 2024 Oct 16]. <https://doi.org/10.1145/3341525.3387396>  121. Ayeni AJ. Principals’ instructional time management and students’ academic performance in secondary schools in Ondo North senatorial district of Ondo State, Nigeria. Journal of Education and Learning (EduLearn). 2020 Feb 1;14(1):123–33. <https://doi.org/10.11591/edulearn.v14i1.14743>  126. Misra RK, Khurana K. Employability Skills among Information Technology Professionals: A Literature Review. Procedia Comput Sci [Internet]. 2017 Jan 1 [cited 2025 Jul 9];122:63–70. <https://doi.org/10.1016/j.procs.2017.11.342>  127. Puwakgahawela A, Mahakalanda I, Wickramasinghe V. Employability Skills of Information Technology Graduates-A Comparison of Expectations, Priorities and Strategies. In: Proceeding of the Open University Research Sessions. The Open University of Sri Lanka; 2023.  128. Carter L. Ideas for adding soft skills education to service learning and capstone courses for computer science students. In: Proceedings of the 42nd ACM Technical Symposium on Computer Science Education [Internet]. New York, NY, USA: Association for Computing Machinery; 2011. p. 517–22. (SIGCSE ’11). Available from: <https://doi.org/10.1145/1953163.1953312>  129. Saadat Z, Alam S, Rehman M. Review of factors affecting gender disparity in higher education. Vol. 8, Cogent Social Sciences. Cogent OA; 2022. <https://doi.org/10.1080/23311886.2022.2076794>  130. Aziz S, Harrizi M, Loutfi A, Zaidoune S. Undergraduates’ Perspectives on the Significance of Soft Skills for Employment: Gender-Based Differences. Journal of Education in Muslim Societies. 2024;6(1):3–31. <http://dx.doi.org/10.2979/jems.00014> |

**Attachment:** Comment 1 of reviewer 2
