# Supplementary material for: Assessing the influence of diverse skills on employability outcomes for IT undergraduates
Source: PLoS One. 2026 Apr 10;21(4):e0346571. doi: 10.1371/journal.pone.0346571 (PMC13068345; doi:10.1371/journal.pone.0346571)
Supplement: Attachment — Submitted filename: Response_to_Reviewers_auresp_2.docx [file pone.0346571.s003.docx]

**Point by point response to the editor-in-chief, and reviewers**

Dear editor-in-chief, and the reviewers,

We would like to express our profound appreciation to the editor-in-chief, and the reviewers for the valuable comments and suggestions made on our manuscript. Your insights have been instrumental in helping us improve the quality and clarity of the paper. We have carefully considered each comment and have revised the manuscript accordingly. All responses and revisions are detailed point by point in the following document.

Please note that the ***line numbers referred to in this document are aligned with the revised manuscript with track changes***.

# Editors' Comments

| **Editor’s comments** | **Response to the comments** |
| --- | --- |
| 1. Please attend to Reviewer #3's comments. Mainly:   - Strengthen your discussion of findings based on the reviewer #3's comments.  - Fix grammar, punctuation errors, and repeated/redundant phrases where relevant.  Also, the text in the figures you supplied in the appendix is difficult to read. Check if you can provide more high-resolution images. | We sincerely thank you for this guidance. In response, we have carefully strengthened the discussion section and literature review by expanding our theoretical explanations in line with Reviewer #3’s suggestions.  The manuscript has been thoroughly checked, and duplicated phrases, spacing inconsistencies, and grammatical errors have been corrected throughout.  The figures have been replaced with improved high-resolution images to enhance visual clarity. |

# Response to Reviewers

| **Reviewers' comments** | **Response to the comments** |
| --- | --- |
| ***Response to Reviewer #3*** | |
| 1. Conceptual overlap between employability, self-efficacy, and technical skills. The HTMT values indicate high correlations between employability, self-efficacy, and technical skills, with some exceeding recommended thresholds. While the authors acknowledge this, the discussion would benefit from a clearer theoretical justification distinguishing these constructs conceptually.   A short paragraph explicitly explaining why employability is not reducible to confidence or skill possession alone would improve interpretability. | We sincerely thank you for the thoughtful evaluation of our manuscript. We greatly appreciate the positive comments on the study’s methodological accuracy and relevance. The suggestions provided have been valuable in strengthening the clarity and theoretical contribution of the paper. In response we explicitly explain in literature review that employability is conceptualized as a broader outcome construct that extends beyond both competence and confidence alone.  **Employability and the Growing Landscape of Skill Sets**  ***(From line number 145-150)***  *“Employability research operates across three conceptual strands: personal strengths that increase employment potential, self-perceived employment opportunities, and actual job transitions as a realization of that potential [28]. Critically, scholars advocating the self-perceived employment approach argue that the personal strengths that increase employment conflict with its origin, therefore treating skills and confidence as synonymous with perceived employability commits a category error [28].”*  ***(From line number 153-169)***  *“While confidence is an internal, affective state, perceived employability is an external, outcome‑oriented judgment that incorporates feedback from recruiters, labor‑market signals, and personal constraints [29]. Consequently, interventions aiming to enhance graduates’ employability must address not only skill development and self‑confidence but also market awareness, networking opportunities, and realistic appraisal of occupational demands [30].*  *Employability is a multi-dimensional construct involving mutual interactions between individual favor and organizational cues, highlighting how interpretation of key events informs individuals and shapes their career paths [32]. Under this framework, perceived employability is negotiated in context that it is coproduced through encounters with labor markets, employers, and professional communities [32]. Findings support the conclusion reached by Soares and Mosquera that perceived employability is related, but distinct and much more than, employability skills and attributes [33,34].”*  ***(From line number 182-190)***  *“For IT undergraduates, perceived employability is shaped by three interrelated factors: Technical competencies and soft skills provide the foundation for self‑assessment, yet the relevance of these skills to current industry demand determines how graduates evaluate their prospects [43]. Networks, mentorship, and internship experiences grant access to hidden opportunities, thereby enhancing perceived employability beyond what skills alone can explain a high level of perceived employability is linked to greater career planning behavior, proactive job search activities, and lower academic anxiety [30,44]. Conversely, low perceived employability can undermine motivation, increase dropout risk, and exacerbate stress [45].”*  ***(From line number 201-204)***  *“Outcomes that improve IT undergraduates’ market awareness, networking opportunities, and realistic skill to job mapping are essential for fostering positive employability perceptions and, consequently, better academic and career outcomes [49].”*  **Mediating Impact of Self-Efficacy**  ***(From line number 452-456)***  *“Research shows that self‑efficacy captures only one dimension of the self‑assessment process, namely the belief in one’s capability to perform specific tasks, whereas perceived employability integrates expectations about demand for those skills, the relevance of one’s qualifications to available positions, and the likelihood of being selected by employers [44,104].”*  The following is a list of DOIs for the citations that were added in the lines above.  [28] van Harten J, de Cuyper N, Knies E, Forrier A. Taking the temperature of employability research: a systematic review of interrelationships across and within conceptual strands. European Journal of Work and Organizational Psychology 2022;31:145–59. <https://doi.org/10.1080/1359432X.2021.1942847>  [29] Fugate M, Kinicki AJ, Ashforth BE. Employability: A psycho-social construct, its dimensions, and applications. J Vocat Behav 2004;65:14–38. <https://doi.org/https://doi.org/10.1016/j.jvb.2003.10.005>  [30] Jackson D, Lambert C, Sibson R, Bridgstock R, Tofa M. Student employability-building activities: participation and contribution to graduate outcomes. Higher Education Research & Development 2024;43:1308–24. <https://doi.org/10.1080/07294360.2024.2325154>  [31] Jackson D, Tomlinson M. Investigating the relationship between career planning, proactivity and employability perceptions among higher education students in uncertain labour market conditions. High Educ (Dordr) 2020;80:435–55. <https://doi.org/10.1007/s10734-019-00490-5>  [32] Dempsey S, Linehan C, Healy M. Negotiating perceived employability as sensemaking in the context of undergraduate work integrated learning. The British Accounting Review 2025:101676. <https://doi.org/https://doi.org/10.1016/j.bar.2025.101676>  [33] Mosquera P, Soares ME. Onboarding: a key to employee retention and workplace well-being. Review of Managerial Science 2025;19:3687–711. <https://doi.org/10.1007/s11846-025-00864-3>  [34] Mainga W, Murphy-Braynen MB, Thompson D, Robins B. Relationship between Perceived Employability and Employability Skills and Attributes among Business Graduates. International Journal for Business Education 2025;167. <https://doi.org/10.61403/2164-2885.1153>  [43] Tung DAO, Huong BUITT. The role of social self-efficacy and psychological capital in IT graduates’ employability and subjective career success. Cogent Soc Sci 2023;9:2204630. <https://doi.org/10.1080/23311886.2023.2204630>  [44] Rothwell A, Arnold J. Self-perceived employability: Development and validation of a scale. Personnel Review 2007;36:23–41. <https://doi.org/10.1108/00483480710716704>  [45] Bernardo AB, García-Gutiérrez V, Esteban M, Maluenda-Albornoz J. Relationship between self-efficacy and university dropout: a systematic review. Front Psychol 2025;Volume 16-2025  [49] [49] Pérez Zúñiga R, Martínez García M, Oliva Ibarra FE. Employability and its relationship with the competency-based approach, teaching methodologies, and assessment in higher education: a systematic review. Front Educ (Lausanne) 2025;Volume 10-2025.  [104] [104] Rothwell A, Jewell S, Hardie M. Self-perceived employability: Investigating the responses of post-graduate students. J Vocat Behav 2009;75:152–61. <https://doi.org/10.1016/j.jvb.2009.05.002> |
| 2. Interpretation of proficiency as a mediator The results show that proficiency mediates the technical skills–employability relationship but not the soft skills–employability relationship. This asymmetry is interesting, yet the discussion treats it somewhat briefly. Expanding the explanation, particularly in relation to UI/UX education and curriculum design, would strengthen the contribution | We are grateful for highlighting this important asymmetry in the mediation findings.  In response, we have expanded the Discussion to more thoroughly.  ***(From line number 849-861)***  *“Proficiency, conceptualized as the demonstrated mastery and practical application of competencies beyond mere acquisition, serves as a critical bridge for technical skills [143]. The finding aligns with competency-based frameworks in IT education, where technical proficiency functions as a controller converting latent knowledge into verifiable performance metrics essential for competitive fields like UI/UX design [144,145]. The mediating role of proficiency in the technical skills and perceived employability pathway reflects the fundamentally graduated, measurable character of technical competencies. In design education, key technical indicators are all domains in which performance exists on a clear scale from novice to expert [146]. Students can therefore appraise their own employability not simply from possessing skills in tools such as Figma or prototyping platforms, but from their subjective assessment of how well they perform with them, suggesting that skill possession alone is an insufficient predictor of perceived employability, and that an intermediate evaluation of proficiency level is what does the explanatory work [24].”*  ***(From line number 872-877)***  *“Among STEM undergraduates, technical proficiency mediated skill to employability perceptions, while soft skill proficiency showed direct effects absent mediation, attributed to assessment biases in self-reports [148,149]. In creative tech fields similar to UI/UX, proficiency in tools like Figma correlated with employability perceptions, but communication mastery did not, as employers prioritize demonstrated teamwork over self-rated aptitude [150–152].”*  ***(From line number 879-884)***  *“These findings carry direct implications for UI/UX curriculum design. Curricular designs should incorporate reflective support, mentor guided supervision, and task role coherence to ensure that learning environments serve as meaningful developmental space where an approach that allows technical proficiency to deepen deliberately while soft skills grow through authentic professional encounter [34].”*  ***(From line number 892-898)***  *“Recent studies highlight that proficiency as the demonstrated mastery and practical application of competencies that acts as a crucial bridge between skill acquisition and employability outcomes, especially in fields such as UI/UX design where technical expertise must be coupled with effective interpersonal abilities [154]. This mediating role underscores that perceived employability hinges on the ability to convert latent knowledge into observable performance, not simply on confidence or the static inventory of skills [24].”*  The following is a list of DOIs for the citations that were added in the lines above.  [34] Mainga W, Murphy-Braynen MB, Thompson D, Robins B. Relationship between Perceived Employability and Employability Skills and Attributes among Business Graduates. International Journal for Business Education 2025;167. <https://doi.org/10.61403/2164-2885.1153>  [143] Alt D, Naamati-Schneider L, Weishut DJN. Competency-based learning and formative assessment feedback as precursors of college students’ soft skills acquisition. Studies in Higher Education 2023;48:1901–17. <https://doi.org/10.1080/03075079.2023.2217203>.  [144] Raj RK, Sabin M, Impagliazzo J, Bowers D, Daniels M, Hermans F, et al. Toward Practical Computing Competencies. Proceedings of the 26th ACM Conference on Innovation and Technology in Computer Science Education V. 2, New York, NY, USA: Association for Computing Machinery; 2021, p. 603–4. <https://doi.org/10.1145/3456565.3461442>.  [145] Yao D, Lin J. Cognitive enhancement through competency-based programming education: a 12-year longitudinal study. Educ Inf Technol (Dordr) 2025;30:20347–83. <https://doi.org/10.1007/s10639-025-13582-w>.  [146] Duggal HK, Lim WM, Khatri P, Thomas A, Shiva A. The state of the art on self-perceived employability. Global Business and Organizational Excellence 2024;43:88–110. <https://doi.org/10.1002/joe.22245>.  [148] Ashlock J, Stojnic M, Tufekci Z. Gender Differences in Academic Efficacy across STEM Fields. Sociological Perspectives 2022;65:555–79. <https://doi.org/10.1177/07311214211028617>  [149] Daun M, Brings J, Obe PA, Stenkova V. Reliability of self-rated experience and confidence as predictors for students’ performance in software engineering. Empir Softw Eng 2021;26:80. <https://doi.org/10.1007/s10664-021-09972-6>.  [150] Palani S, Ledo D, Fitzmaurice G, Anderson F. ”I don’t want to feel like I’m working in a 1960s factory”: The Practitioner Perspective on Creativity Support Tool Adoption. Proceedings of the 2022 CHI Conference on Human Factors in Computing Systems, New York, NY, USA: Association for Computing Machinery; 2022. <https://doi.org/10.1145/3491102.3501933>.  [151] Mulifah M, Anifah L, Tjahjanto I. The Impact of Technical Skills on Students’ Employability Skills in the Creative Industry Era. Edu Cendikia: Jurnal Ilmiah Kependidikan 2024;4:1215–23.  [152] Sosa R, Rajusha R, Hunting A. Landing your first job in Creative Technologies: Soft skills as Core skills. Des Technol Educ 2022;27:65–81. |
| 3. Gender moderation effects The moderation results for gender are statistically significant and relevant. However, the manuscript would benefit from a more cautious interpretation, explicitly noting that the study examines perceived employability rather than actual hiring outcomes. This clarification would help avoid overgeneralization. | We thank the reviewer for this thoughtful recommendation regarding interpretive caution. In response, we have explicitly clarified that the study examines perceived employability rather than actual hiring outcomes.  **Employability and the Growing Landscape of Skill Sets**  ***(From line number 153-169)***  *“While confidence is an internal, affective state, perceived employability is an external, outcome‑oriented judgment that incorporates feedback from recruiters, labor‑market signals, and personal constraints [29]. Consequently, interventions aiming to enhance graduates’ employability must address not only skill development and self‑confidence but also market awareness, networking opportunities, and realistic appraisal of occupational demands [30].*  *Employability is a multi-dimensional construct involving mutual interactions between individual favor and organizational cues, highlighting how interpretation of key events informs individuals and shapes their career paths [32]. Under this framework, perceived employability is negotiated in context that it is coproduced through encounters with labor markets, employers, and professional communities [32]. Findings support the conclusion reached by Soares and Mosquera that perceived employability is related, but distinct and much more than, employability skills and attributes [33,34].”*  ***(From line number 182-190)***  *“For IT undergraduates, perceived employability is shaped by three interrelated factors: Technical competencies and soft skills provide the foundation for self‑assessment, yet the relevance of these skills to current industry demand determines how graduates evaluate their prospects [43]. Networks, mentorship, and internship experiences grant access to hidden opportunities, thereby enhancing perceived employability beyond what skills alone can explain a high level of perceived employability is linked to greater career planning behavior, proactive job search activities, and lower academic anxiety [30,44]. Conversely, low perceived employability can undermine motivation, increase dropout risk, and exacerbate stress [45].”* |
| 4. Minor language and presentation issues There are several instances of duplicated phrases, inconsistent spacing, and minor grammatical errors (for example, repeated sentences in the literature review and discussion sections). These do not undermine the study but should be corrected for readability, especially as PLOS ONE does not copyedit accepted manuscripts. | We appreciate this careful reading of the manuscript.  All duplicated phrases, spacing inconsistencies, and minor grammatical issues have been corrected throughout the document.  We thank the reviewer for noting these details, and we have ensured that the revised version reflects improved clarity and consistency. |
